# Polyphenolic Composition, Mineral Profile, and Biological Activities in Different Organs of Alpine Woundwort—Insights into Antioxidant and Enzyme Inhibitory Potential

**DOI:** 10.3390/ph18050674

**Published:** 2025-05-01

**Authors:** Sabina Lachowicz-Wiśniewska, Ireneusz Ochmian, Jan Oszmiański, Rafał Wiśniewski, Małgorzata Bernatek, Paweł Rubiński, Daniela De Vita

**Affiliations:** 1 Department of Medicine and Health Science, University of Kalisz (Calisia University), plac Wojciecha Bogusławskiego 2, 62-800 Kalisz, Poland; j.oszmianski@upwr.edu.pl (J.O.); m.bernatek@uniwersytetkaliski.edu.pl (M.B.); p.rubinski@uniwersytetkaliski.edu.pl (P.R.); 2 Department of Production Engineering, Wroclaw University of Economics and Business, Komandorska 118/120, 53-345 Wrocław, Poland; rafal.wisniewski@ue.wroc.pl; 3 Department of Horticulture, West Pomeranian University of Technology in Szczecin, 71-434 Szczecin, Poland; ireneusz.ochmian@zut.edu.pl; 4 Dipartimento di Biologia Ambientale, Università di Roma “La Sapienza”, Piazzale Aldo Moro 5, 00185 Rome, Italy; daniela.devita@uniroma1.it

**Keywords:** polyphenols, mineral composition, biological activity, antioxidants, digestive enzymes, Alpine woundwort

## Abstract

**Background:** *Stachys alpina* is a medicinal plant from the Lamiaceae family whose biological potential remains poorly explored. **Methods:** The aim of this study was to comprehensively assess the pol-yphenol profile, macro- and microelement composition, and the antioxidant, an-ti-diabetic, and anti-obesity activities of various plant organs (leaves, flowers, stems, and roots). **Results:** The leaves and flowers exhibited the highest concentration of phenolic compounds, while anthocyanins were detected exclusively in the flowers (215.05 mg/100 g dry matter (dm)) and constituted 3% of the total polyphenols. Verbas-coside and chlorogenic acid were the most abundant polyphenols, reaching 4618.88 and 3277.83 mg/ 100 g dm in the leaves. The highest ABTS and FRAP scavenging activity was observed in leaves (19.30 and 7.62 mmol TE/g dm, respectively). Principal component analysis demonstrated a strong correlation between polyphenol content and antioxidant activity (ABTS-r= 0.87 and FRAP-r = 0.90), which was further confirmed by Pearson’s correlation coefficients. The study also highlighted the significant impact of mineral composition on biological activity—calcium and magnesium dominated in stems (10,100 and 3900 mg/kg) and in roots (9200 and 3100 mg/kg), supporting the functioning of an-tioxidant enzymes, while zinc and manganese in leaves (89.43 and 155.33 mg/kg) con-tributed to intense metabolic processes. **Conclusions:** *S. aplina* could serve as a valuable source of natural antioxidants and enzyme inhibitors associated with glucose and lipid metabolism, suggesting its promising application in the prevention and management of metabolic disorders..

## 1. Introduction

The Stachys genus, comprising over 300 species within the Lamiaceae family, represents one of the most chemically diverse and pharmacologically promising plant groups. Many species within this genus have been traditionally utilized in herbal medicine for their broad spectrum of therapeutic effects, particularly their anti-inflammatory, antioxidant, antimicrobial, and metabolic regulatory properties [1,2]. These properties are primarily attributed to their rich content of secondary metabolites, including polyphenols, flavonoids, iridoids, and phenylethanoid glycosides, which have been reported to exhibit significant health benefits [3,4].

Among the various Stachys species, *Stachys alpina* L. has garnered increasing interest due to its occurrence in mountainous ecosystems and its adaptation to extreme environmental conditions, which may contribute to its unique phytochemical composition and bioactivity. However, despite its potential, the chemical profile and biological activities of *S. alpina* remain largely underexplored [3,4,5,6]. Unlike well-characterized medicinal plants, only a few studies have assessed its polyphenolic profile, essential mineral composition, and enzymatic inhibitory activities in relation to human health and disease prevention. *S. alpina* (common name: Alpine woundwort) typically grows up to 100 cm in height, and is characterized by serrated ovate leaves and purplish-to-violet flowers arranged in whorls. It naturally occurs in humid, shaded, mountainous forests across Central and Southern Europe, particularly in the Alps, the Carpathians, and the Balkans, and is also found sporadically in parts of West Asia [7,8,9,10]. Due to its ecological niche, *S. alpina* is not widely cultivated on a commercial scale; instead, it is collected from wild populations or cultivated in small experimental or botanical gardens. Data on its large-scale cultivation and annual production are currently lacking in the literature, likely due to its underutilized status and the relatively recent interest in its bioactive potential [4,10].

*S. alpina* is a promising source of polyphenolic compounds—particularly phenolic acids, flavonoids, and phenylethanoid glycosides—which exhibit strong antioxidant and neuroprotective properties [3,4]. These compounds help to counteract oxidative stress, a key factor in the development of cardiovascular diseases, neurodegenerative disorders, metabolic syndrome, and certain cancers [3].

Recent research highlights the potential of *S. alpina* extracts in managing metabolic disorders through the inhibition of digestive enzymes such as α-amylase, α-glucosidase, and pancreatic lipase, which are involved in carbohydrate and lipid metabolism [4,5,6,9,10]. This positions the species as a candidate for the development of nutraceuticals aimed at blood glucose control, lipid regulation, and cognitive support. The plant’s content of essential minerals (e.g., magnesium, calcium, zinc, manganese) may also contribute to enzymatic balance and metabolic homeostasis [2,4]. Additionally, bioactive constituents such as phenylethanoid glycosides may support applications in anti-inflammatory, antimicrobial, and neuroprotective therapies [2,3]. Given the global burden of non-communicable diseases and growing demand for natural health products, *S. alpina* offers an avenue for plant-based interventions, with potential societal and economic benefits through its inclusion in sustainable health-promoting formulations [10,11,12]. Although not yet used in mainstream pharmaceutical production, *S. alpina* has shown potential for use in the development of dietary supplements and herbal products targeting metabolic syndrome, neurodegenerative diseases, and chronic inflammation. Based on their identified bioactive compounds, plant extracts—particularly those derived from the aerial parts (leaves and flowers)—can be formulated into capsules, tablets, or powder mixtures. These forms are suitable for use as nutritional supplements, especially those aimed at blood glucose regulation, antioxidant support, or mild anti-inflammatory action. Traditional use also suggests the potential development of herbal teas and topical preparations from *Stachys* species, though clinical validation is needed [4].

To address the current gaps in knowledge, this study provides a comprehensive analysis of the phytochemical composition, mineral profile, and biological activities of different organs of *S. alpina*. The main objectives include profiling the polyphenolic composition of *S. alpina* using advanced chromatographic techniques, such as UPLC-PDA-MS/MS, to identify bioactive compounds responsible for its therapeutic properties. Additionally, the study aims to assess the enzyme inhibitory potential of *S. alpina* extracts against key metabolic enzymes involved in glucose and lipid metabolism, highlighting their relevance for obesity and diabetes management. Also, the antioxidant activity of different plant organs is evaluated to determine their capacity to combat oxidative stress and prevent cellular damage. Finally, the study investigates the relationship between mineral composition and bioactivity, considering the role of essential elements in metabolic regulation and enzymatic function.

## 2. Results and Discussion

### 2.1. Mineral Compounds and Nitrates and Nitrites

The content of macroelements in different parts of *S. alpina* —roots, stems, leaves, and flowers—exhibits significant differences, resulting from these organs’ distinct metabolic and structural functions [Table 1]. The analysis results indicate that mineral elements primarily accumulate in leaves and stem, emphasizing their crucial role in photosynthetic processes, growth, and the maintenance of structural integrity.

The leaves demonstrated the highest concentration of macroelements, particularly nitrogen, phosphorus, and potassium. The high nitrogen content reflects its importance in protein and chlorophyll synthesis, while phosphorus, as a key component of nucleotides and ATP, supports intensive energy metabolism. Potassium plays a significant role in water balance regulation and maintaining the osmotic potential of cells. The stems were characterized by a high calcium and magnesium content. Calcium is essential for cell wall stabilization and signal transduction, whereas magnesium, as an enzymatic cofactor, is indispensable for the functioning of the photosynthetic apparatus. The roots exhibited moderate levels of calcium and magnesium, reflecting their role in mineral storage and transport. The flowers had the lowest concentrations of most macroelements, which can be attributed to their generative function, unrelated to intensive vegetative metabolism. The exception to this was phosphorus, whose elevated levels indicate its significance in energy processes associated with flowering and seed formation.

The microelement content in *S. alpina* also exhibited substantial variability depending on the plant organ [Table 1]. Iron, a key element in photosynthetic processes and chlorophyll synthesis, reached its highest concentration in the leaves, and its concentration was significantly lower in the roots. Manganese, as a cofactor of numerous enzymes involved in energy metabolism and reactive oxygen species neutralization, was most concentrated in the leaves and stems. In contrast, it had only a low content in the roots. Zinc, crucial for DNA polymerase function and oxidative stress protection, exhibited the highest concentration in the flowers, which can be linked to its role in generative processes, while its lowest content was recorded in the roots. Copper, essential for the activity of enzymes involved in electron transport and lignin metabolism, dominated in the leaves and stems, reaching its lowest levels in the roots. Trace amounts of nickel and cadmium were detected in all plant organs, without significant distribution differences. Selenium, however, was most concentrated in the flowers, suggesting its possible role in oxidative stress protection and support of developmental processes in generative organs.

The results of the macroelement distribution analysis in *S. alpina* align with general trends observed in other medicinal plants. As reported in the literature, the leaves represent the most metabolically active part of the plant, explaining their high nitrogen, phosphorus, and potassium content. In contrast, stems, as supporting and transport structures, contain higher amounts of calcium and magnesium, which are necessary for the maintenance of cell wall integrity. Studies by Arceusz et al. [13,14] have demonstrated that the macroelement content in medicinal plants can vary significantly depending on species and environmental conditions, yet the general pattern of element distribution remains similar to that observed in *Stachys caroliniana* Nelson and Rayner [8]. A review by Baloch [15] highlighted that the mineral composition of medicinal plants exhibits strong variation depending on habitat conditions and genotype, which may explain the differences in mineral composition observed among different *S. alpina* populations. Additionally, research published by Szentmihalyi et al. [16] indicated that the microelement content in medicinal plants also depends on the species and plant part, influencing their pharmacological properties. Similarly, the analysis of trace elements in five popular medicinal plants in Iran revealed varying microelement concentrations depending on the species and the analyzed plant part [17]. Meanwhile, a report presented by Bouasla et al. [18], assessing the mineral content in *S. marrubiifolia* leaves, found that the macroelement concentrations were 286,000 mg/kg for magnesium, 104,000 mg/kg for calcium, 180,000 mg/kg for potassium, and 11,400 mg/kg for phosphorus. The phosphorus content was highly variable between species, with a difference as high as 191-fold. The concentrations of potassium and calcium in *Stachys marrubiifolia* Viv. leaves were approximately 5.6 and 5.2 times lower, respectively, compared to those in *S. alpina* leaves, while the magnesium content remained at a similar level in both species.

The microelement content in *S. marrubiifolia* leaves was as follows: zinc—1860 mg/kg, iron—13,000 mg/kg, manganese—500 mg/kg, copper—400 mg/kg, and selenium—1.9 mg/kg [18]. Compared to *S. alpina* leaves, the concentrations of iron and zinc were slightly higher, by approximately 1.45- and 1.14-fold, respectively (i.e., *S. alpina* contained 0.69 and 0.9 times lower amounts of these elements). In contrast, the manganese content was significantly higher in *S. alpina*, being 31 times greater than in *S. marrubiifolia*, indicating a pronounced difference in favor of *S. alpina*. The copper content was also notably higher in *S. alpina*, by a factor of 3.1.

Selenium and nickel were detected only in *S. marrubiifolia* (1.9 mg/kg and 4.0 mg/kg, respectively), while their concentrations in *S. alpina* remained below the detection limit (<1 mg/kg), suggesting at least a 2-to-4-fold difference. The cadmium content was three times higher in *S. alpina* (3.0 mg/kg) compared to *S. marrubiifolia* (1.0 mg/kg) [18].

The detected nickel and cadmium contents were at the same level [18]. Additionally, *S. marrubiifolia* leaves were found to contain cobalt (2.0 mg/kg), sulfur (1,536,400 mg/kg), and arsenic (<0.1 mg/kg) [18]. To our knowledge, this is the first report providing a comprehensive analysis of macro- and microelements in different plant parts of *S. alpina*.

Thus, leaves constitute the most metabolically active fraction of the plant, rich in macroelements such as nitrogen, phosphorus, and potassium, as well as microelements such as iron, manganese, and copper. Stems exhibit moderate macro- and microelement concentrations, reflecting their transport-related and structural functions. Roots accumulate relatively low amounts of most elements, primarily serving storage and nutrient-absorbing functions. Flowers are distinguished by elevated zinc and selenium concentrations, which may be related to their role in generative processes and environmental stress protection. These differences highlight the specialized functions of distinct plant fractions and their potential applications in various biological and industrial contexts.

The nitrate (NO_3_⁻) and nitrite (NO_2_⁻) content in different organs of *S. alpina* exhibited significant variability, which may have resulted from specific metabolic mechanisms occurring within the plant. The highest concentration of nitrates was recorded in the stems (552.30 mg/kg) [Table 1], which may be attributed to their role as conductive structures involved in nitrogen transport and transformation—processes crucial for physiological functions, including photosynthesis. The flowers exhibited moderate NO_3_⁻ levels (187.50 mg/kg), whereas the nitrate content in the leaves and roots was 5.1 and 5.3 times lower than in the stems, respectively. A similar trend was observed for the nitrite (NO_2_⁻) content, with the highest concentration also detected in the stems (1.80 mg/kg), followed by the flowers (1.20 mg/kg). The lowest values were noted in the leaves and roots, which contained 2.6 and 1.5 times less NO_2_⁻ than the stems, respectively [19].

Nitrates and nitrites are natural plant metabolites that play a crucial role in nitrogen metabolism. However, excessive intake of these compounds can have serious health consequences, particularly due to their conversion into nitrosamines—compounds with confirmed carcinogenic potential. This conversion occurs under low-pH conditions in the stomach and through the action of intestinal microbiota. The European Food Safety Authority (EFSA) has established the acceptable daily intake (ADI) for nitrates at 370 mg/kg body weight, whereas for nitrites, this value is set at 6.0 mg/kg body weight. Exceeding these limits may be associated with the risk of methemoglobinemia and potential involvement in carcinogenic processes [20].

The analysis of the obtained results indicates that the nitrate content in the stems and flowers of *S. alpina* raises certain concerns regarding the consumption of large quantities of this raw material, particularly in diets rich in leafy vegetables, which are also significant sources of nitrates. However, these values remain within acceptable limits for herbaceous plants, according to data from the literature. Studies conducted by Santamaria [21] have revealed that nitrate levels in leafy vegetables such as spinach and lettuce can reach 250,000–300,000 mg/kg, which significantly exceeds the levels found in *S. alpina* L. This suggests that while the nitrate content in the stems of the studied plant is relatively high, it remains well below the values recorded in common leafy vegetables.

Similar patterns apply to nitrite content. Research published in the *Journal of Food Composition and Analysis* confirms that the concentrations of these compounds in herbaceous plants and medicinal herbs are generally low, aligning with the values obtained for the leaves and roots of *S. alpina.* Scientific research highlights that nitrate and nitrite levels in plants depend on multiple factors, including the species, the plant organ, and environmental and agronomic conditions. Studies indicate that the highest concentrations of NO_3_⁻ and NO_2_⁻ are typically found in leaves, due to their intensive involvement in metabolic processes and the accumulation of nitrates as a reserve nitrogen form [21]. In a comparative context, medicinal plants such as marjoram (*Origanum majorana* L.) [22] have been reported to contain significantly higher nitrate levels than cultivated vegetables. Regarding *S. alpina,* the obtained values fall within the range reported for other species within the Lamiaceae family, suggesting that moderate consumption of this plant does not pose a significant health risk.

### 2.2. Identification and Quantification of Polyphenolic Compounds

Analysis of the chemical composition of *S. alpina*, performed using ultra-performance liquid chromatography coupled with a photodiode array detector and tandem mass spectrometry (UPLC-PDA-MS/MS (Waters Corporation, Milford, MA, USA)), enabled the identification of 58 polyphenolic compounds. Their distribution varied among different plant organs: the leaves contained 54 compounds, the flowers contained 56, the stems contained 51, and the roots contained 38. Furthermore, UPLC analysis identified five groups—phenylethanoid glycosides (PhGs), iridoids, phenolic acids (PAs), flavonols and flavones (F&Fs), and anthocyanins (ANTs)—comprising a total of 22 compounds. The identification of these compounds was based on their molecular mass, MS/MS fragmentation patterns, retention time, and comparison with data from the literature and available analytical standards [23,24,25,26,27,28,29,30,31,32,33,34,35,36,37,38,39].

The analysis of the obtained data indicates a diversified mass distribution of different groups of chemical compounds, which is evident both in their mass spectral characteristics (MS/MS) and retention times. Phenolic acids, such as 1-caffeoylquinic acid (*m*/*z* 353), 4-caffeoylquinic acid (*m*/*z* 353), and 5-caffeoylquinic acid (*m*/*z* 353), exhibit relatively low molecular weights, reflecting their simpler chemical structure. Most of these compounds fall within the *m*/*z* 300–400 range, confirming their role as primary bioavailable constituents. Higher molecular masses, such as *m*/*z* 515 in the case of 3,4- and 3,5-dicaffeoylquinic acids, indicate the presence of additional functional groups, which may enhance the compounds’ antioxidant properties [25].

Anthocyanins, represented by compounds such as cyanidin 3-glucoside (*m*/*z* 449) and peonidin 3-*O*-glucoside (*m*/*z* 463), exhibit slightly higher molecular weights than phenolic acids, due to the presence of glycosyl groups. Cyanidin 3-malonylglucoside (*m*/*z* 535) and peonidin 3-malonylglucoside (*m*/*z* 549) possess even higher masses, resulting from malonyl group modifications, which increase their molecular weight. These compounds, due to their higher molecular mass, may exhibit more complex biological properties, including enhanced pigment stability under different environmental conditions [23,24,25,26].

Iridoids are characterized by molecular masses higher than those of phenolic acids and anthocyanins. Verbascoside (*m*/*z* 623) and its isomers (also *m*/*z* 623) dominate this group, with their widespread presence observed in the analyzed samples. Notably, stachysoside A (*m*/*z* 755) and forsythoside B (*m*/*z* 755) exhibit even higher molecular weights, due to their complex structural arrangement, including the presence of sugar moieties and phenolic residues [25,26,27,28,29].

Flavones and flavonols exhibit some of the highest molecular masses in this dataset, reflecting their complex chemical structures. For instance, luteolin 7-*O*-[6‴-*O*-acetyl]-allosyl-(1→2)-glucoside (*m*/*z* 651) and chrysoeriol 7-*O*-[6‴-*O*-acetyl]-allosyl(1→2)-glucoside (*m*/*z* 665) fall within the 600–700 *m*/*z* range, due to acetylation and the presence of sugar residues. Even higher molecular masses, such as *m*/*z* 797 in the case of chrysoeriol 7-*O*-[6‴-*O*-acetyl]-allosyl(1→2)-glucoside-pentoside, suggest highly complex structural arrangements and potentially unique biological properties. Other compounds, such as leonoside B (*m*/*z* 783) and isoscutellarein 7-*O*-[6‴-acetylallosyl-(1→2)]-glucopyranoside (*m*/*z* 651), also fall within the higher mass range, highlighting their chemically intricate nature. The presence of multiple acetyl and glycosyl groups in their structure further increases their molecular weight [25,26,27,28,29,36,40].

The analysis reveals that the compounds numbered 1, 4, 9, 24, 27, 28, 30, 32, 36, 37, 47, 49, 52, 54, 55, 57, and 58 (Table 2) have previously been identified and described in *S. alpina* [23,40]. However, the remaining polyphenolic compounds identified in different components of *S. alpina* are reported for the first time here, based on data from other botanical sources. The compounds numbered 2, 10, 11, 13, 14, 17, 19–21, 23, 29, 31, 33–35, 39–41, 45, and 56 (Table 2) have been previously characterized and documented in other Stachys species [23,25,26,28,29].

Furthermore, caffeoylglucose (*m*/*z* 341) has been previously identified in *Sideritis sipylea* Boiss. [30], and caffeoylquinic acid (*m*/*z* 353) has been reported in carrots (*Daucus carota* L.) [32]. Two compounds, methoxycinnamic acid hexoside (*m*/*z* 339), were characterized based on data from medicinal plants in Türkiye [33]. The compounds identified as 3-*O*-*p*-coumaroylquinic acid (*m*/*z* 337) and chryseriol 7-*O*-[6‴-*O*-acetyl]-allosyl(1→2)-glucoside (*m*/*z* 665) were identified based on the fragmentation pathways of compounds reported in green Robusta and Arabica coffee beans [34].

Similarly, the compounds with molecular masses *m*/*z* 535 and 549 were identified within the anthocyanin group as cyanidin 3-malonylglucoside and peonidin 3-malonylglucoside, based on data from *Lactuca sativa* L. leaves [35] and red *Allium cepa* L. [36], respectively. The compound with a molecular mass of *m*/*z* 397, tentatively named 5-Sinapoylquinic acid, was identified based on its fragmentation pathway described in *Gardeniae Fructus* [37].

Further characterization of compounds included 4′*-O-*methylisoscutellarein 7*-O-*[6‴*-O-*acetyl]-allosyl-(1→2)-[6′′*-O-*acetyl]-glucoside (*m*/*z* 707), chryseriol 7*-O-*[6‴*-O-*acetyl]-allosyl(1→2)-glucoside (*m*/*z* 665), apigenin 7*-O-*[6‴*-O-*acetyl]-allosyl(1→2)-glucoside (*m*/*z* 635), luteolin 7*-O-*[6‴*-O-*acetyl]-allosyl-(1→2)-[6′′*-O-*acetyl]-glucoside (*m*/*z* 693), and samioside (*m*/*z* 755), which were tentatively identified based on MS/MS data reported for mountain tea from the Balkans [38]. Additionally, the compound tentatively named chrysoeriol-glucoside (*m*/*z* 461) was characterized based on identification data from *Salvia* L. (Lamiaceae) [39]. Lastly, two compounds named leonoside B (*m*/*z* 783) were identified based on fragmentation pathways described in *Leonurus cardiaca* L. [31].

Table 2 presents the polyphenolic compound contents in different organs of *S. alpina* L., including the flowers, leaves, stems, and roots, determined through UPLC-PDA-MS/MS analysis. The total polyphenol content was 31,413 mg/100 g dry matter (DM), with the highest concentration recorded in the leaves and the lowest in the stems and roots. These findings align with those of Vundać et al. [23], who reported the total polyphenol content in *S. alpina* at 5.74%, compared to *S. officinalis* (L.) Trevis. (6.75%), *S. palustris* L. (10.80%), *S. salviifolia* Ten. (6.75%), and *S. sylvatica* L. (3.37%). These values are 3–4 times lower than those observed in the present study. Similarly, in research conducted by Karioti et al. [40], the polyphenol content in various Stachys subspecies ranged from 53.34 g/100 g DM in *S. atherocalyx* K.Koch (Treska Valley, North Macedonia) to 9.58 g/100 g DM in *S. aplina* subsp. *dinarica* Murb. (Mt. Jahorina, Bosnia and Herzegovina). The results obtained in the present study fall within this range, confirming significant variability in polyphenol content depending on the species and ecological location.

A similar trend was observed in the analyses conducted by Lachowicz-Wiśniewska et al. [26] for *S. palustris* L., where the polyphenol content was recorded as 9252 mg/100 g dmDM in the leaves, 8544 mg/100 g dmDM in the flowers, 4932 mg/100 g dmDM in the stems, and 1622 mg/100 g dmDM in the roots. The polyphenol content in *S. alpina* flowers was comparable, whereas it was 1.7 times higher than in *S. palustris* in the leaves, 2.2 times lower in the roots, and 1.3 times higher in the stems. Despite these differences, the accumulation pattern of polyphenolic compounds remained consistent, highest in the leaves and lowest in the roots [26]. The lowest polyphenol content was found in *Stachys scardica* Griseb. extracts, where in situ plants contained 203 μg GA/mg extract, ex vitro plants contained 175.5 μg GA/mg extract, and in vitro plants contained 41.9 μg GA/mg extract [41]. For *Stachys cretica* subsp. *mersinaea* (Boiss.) Rech. [42,43] and *S. cretica* subsp. *kutahyensis* Akçiçek [44], the total polyphenol content was reported as 83.57 mg GA/g, comparable to the content found in *S. alpina* flowers, but half of the content found in the leaves; meanwhile, 41.17 mg GA/g was observed in the stems and roots, which was 2 and 4 times lower than in the flowers and leaves, respectively.

**Figure 1:**
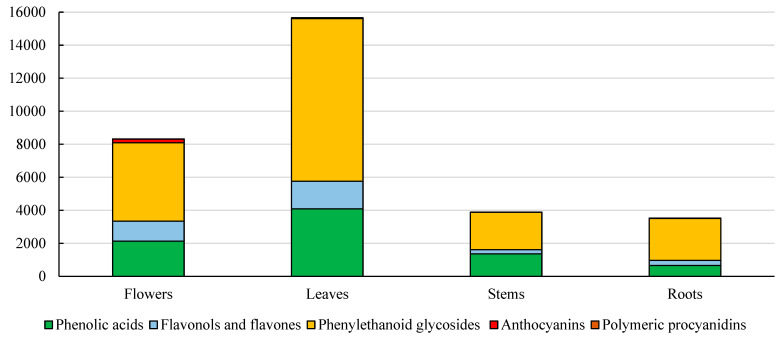
Total content of individual groups of polyphenolic compounds [mg/100 g dm].

The main polyphenolic classes identified in *S. alpina* extracts (from flowers, leaves, stems, and roots) were as follows: phenylethanoid glycosides (comprising an average of 63% of all polyphenolic compounds) > phenolic acids (26%) > flavonols and flavones (10%) > anthocyanins (3%) > polymeric procyanidins (<0.7%) (Figure 1). Compared to *S. palustris*, in which hydrolyzable tannins were the predominant polyphenol group [26], *S. alpina* exhibited a distinctly different composition. These findings indicate substantial differences between the two species, as in *S. palustris*, hydrolyzable tannins represented the dominant fraction of polyphenolic compounds [26,45,46].

**Figure 2:**
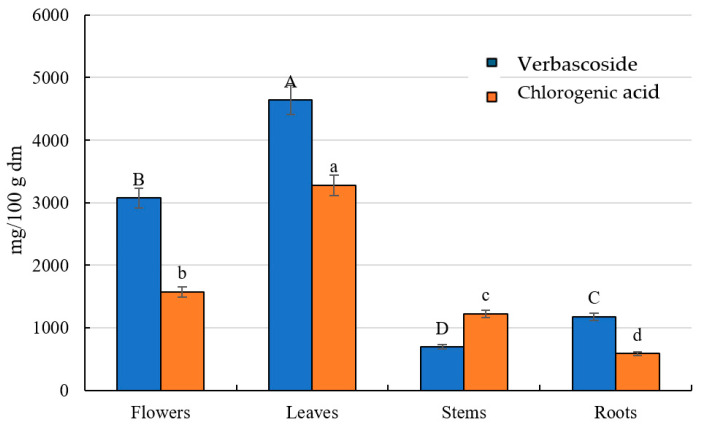
Content of verbascoside and chlorogenic acid as most abundant polyphenols. a–d; A–D: means ± SD followed by different letters within same line represent significant differences (*p* < 0.05).

The content of phenolic acids in different organs of *S. alpina* ranged from 19% in the roots to 35% in the stems, relative to the total polyphenol content. The highest concentration was recorded in the leaves, where the levels were 2, 3, and 6 times higher than in the flowers, stems, and roots, respectively. The predominant compound was 5*-O-*caffeoylquinic acid (chlorogenic acid), accounting for 74% of the total phenolic acids in flowers and up to 90% of those in the stems and roots (Figure 2). These values align with the findings of Karioti et al. [40], Bahadori et al. [43], and Benabderrahim et al. [44]. In contrast, in *S. cretica* subsp. *mersinaea,* the dominant phenolic acids were gallic acid and trans-cinnamic acid [42]. The chlorogenic acid content in various *Stachys* species ranged from 3.17% DM in *S. alpina* subsp. *dinarica* from Mt. Jahorina (Bosnia and Herzegovina) to 12.57% DM in *S. zepcensis* Formánek [40], reaching 29,943.79 µg/g dmDM in *S. cretica* subsp. *kutahyensis* [44], comparable to the values obtained in the present study. In *S. cretica* subsp. *smyrnaea*, the chlorogenic acid content was 426 µg/g DMdm [43], while in *S. tmolea* Boiss., it reached 1120.14 µg/g DM [47], and in *Stachys cretica* subsp. *mersinaea*, it was 350 µg/g DM [42]. Moreover, the content of chlorogenic acid in decoctions and infusions of S. recta was 2.84 and 3.49 mg/g, respectively [24]. Notably, the concentrations of 5-sinapoylquinic, 3,4-dicaffeoylquinic, and 3,5-dicaffeoylquinic acids were 1.3, 1.7, and 1.5 times higher compared to their levels in the leaves. According to Vundać et al. [23], the total phenolic acid content in *S. aplina* L. was 2.01%, whereas in *S. officinalis* L., *S. palustris* L., *S. salviifolia* L., and *S. sylvatica*, it was 2.67%, 6.37%, 2.59%, and 2.41%, respectively—approximately four times lower than in *S. aplina* L. from Poland. In the analyzed components of *S. palustris* [26], chlorogenic acid was not detected, but 3,4-dicaffeoylquinic and 3,5-dicaffeoylquinic acids were identified at levels comparable to those found in *S. alpina* roots [26]. Additionally, *S. tmolea* contained *p*-coumaric acid at a concentration of 3 µg/g DM [47], similar to the findings of the present study. However, this species exhibited an opposite accumulation trend, with the highest phenolic acid concentration recorded in the roots and the lowest in the stems. In contrast, *S. alpina* demonstrated the highest accumulation of these compounds in the flowers, particularly 3,4-dicaffeoylquinic acid [26].

Phenylethanoid glycosides (PhGs) constituted between 57% of the total polyphenolic content in the flowers and stems and 72% in the roots of *S. alpina* [40]. The highest concentration was observed in the leaves, where the PhG levels were 2-, 4-, and 4-fold higher compared to those in the flowers, stems, and roots, respectively. Among the analyzed compounds, verbascoside and satchysoside A were the most abundant, accounting for 30–65% of the total PhGs in the stems and flowers and 19–39% of those in the flowers and stems (Figure 2). The total tannin content across different Stachys species ranged from 0.64% to 2.03% dm in *S. sylvatica* and *S. palustris* [23]. In another study, the PhG levels in various *Stachys* species ranged from 1.09% to 32.90% dm, with the lowest content found in *S. zepcensis* and the highest in *S. atherocalyx* from Treska Valley, North Macedonia [40]. The PhG concentration in decoctions and extracts of *S. recta* was 2.55 and 3.21 mg/g, respectively [24]. In contrast, in different parts of *S. palustris*, the highest PhG concentration was noted in the roots, reaching 209.5 mg/100 g dm, which was, on average, 34 times higher than in the other plant components [26]. This finding is inversely proportional to the results obtained for *S. alpina*, where the lowest PhG levels were recorded in the roots and stems. In *S. tmolea* and *S. cretica* subsp. *kutahyensis*, only verbascoside was detected among the PhGs, with the concentrations being 7 times higher and 63 times lower than in *S. aplina* L., respectively [44,47]. The biological activity of verbascoside includes anti-inflammatory, anti-tumor, and anti-radical properties [26,47].

Flavones and flavonols (F&Fs) represented approximately 10% of all polyphenolic compounds, with the highest concentration in the leaves (1673 mg/100 g). The levels in the flowers, stems, and roots were 1-, 7-, and 5-fold lower, respectively. Among flavones, chrysoeriol 7*-O-*[6‴*-O-*acetyl]-allosyl(1→2)-glucoside was the most abundant, contributing 31% in the leaves and up to 46% in the roots within the F&F class. Apigenin 7*-O-*[6‴*-O-*acetyl]-allosyl(1→2)-glucoside was also predominant in the leaves (223.23 mg/100 g), whereas its concentration in the flowers, stems, and roots was 5-, 31-, and 32-fold lower, respectively. The total F&F content in extracts of *Stachys cretica* subsp. *mersinaea* was 50.10 mg RE/g [43], which was three times higher than in the leaves analyzed in the present study. In *S. tmolea*, the content was 4.98 mg Q/g dm [47], a value comparable to those in the stems and roots, but 2.5- and 3.5-fold lower than in the flowers and leaves. In *S. cretica* subsp. *kutahyensis*, the content was 40.24 mg Q/g dm [44], which was 2.4 times higher than in the leaves. A detailed chromatographic analysis revealed the presence of quercetin, kaempferol, hesperidin, and apigenin in extracts of *Stachys cretica* subsp. *mersinaea*, with a total content of 14,161 µg/g dm [42], a concentration similar to that in the flowers and 1.3 times lower than in the leaves. Further analysis of *S. tmolea* extracts confirmed the presence of luteolin, hesperidin, hyperoside, apigenin, and pinoresinol, with a concentration of 171.37 µg/g dm [47], which was 14 to 92 times lower than in the stems and leaves of *S. aplina* L. In *S. cretica* subsp. *kutahyensis* [44], these compounds were detected at a level of 520.09 µg/g dm, which was 5-to-32-fold lower than in the stems and leaves. Similarly, *S. scardiaca* contained 41 µg Q/mg extract in in vitro conditions and 34.6 µg Q/mg extract in situ [41]. The identified compounds exhibit well-documented anti-inflammatory, antioxidant, and anticancer activities [26,47].

Anthocyanins were detected exclusively in the flowers of *S. alpina,* where their total content reached 215.05 mg/100 g, constituting 3% of the total polyphenolic compounds. The predominant anthocyanin was cyanidin 3-malonylglucoside, accounting for 76% of the total anthocyanin fraction. In contrast, in *S. palustris*, the dominant anthocyanin was malvidin 3*-O-*diglucoside, whose concentration was 10.7 times lower than in *S. alpina* flowers [26]. Additionally, in extracts from *S. marrubiifolia* and *Lamium flexuosum* Ten., the total anthocyanin content, expressed as cyanidin 3*-O-*glucoside equivalent, was 5.37 and 17.25 mg/g extract, respectively, which was 2- and 8-fold higher than the content in *S. alpina* flowers [43].

The smallest group among the polyphenols comprised procyanidin polymers, representing between 0.2% and 1% of the total polyphenolic content in flowers and roots, respectively. The highest concentration of these compounds was noted in the leaves, where the levels were 2-, 3-, and 5-fold higher compared to those in the roots, flowers, and stems, respectively. The average polymerization degree was 1.5, with the highest values observed in the leaves and the lowest in the roots. The procyanidin content in extracts from different *S. palustris* fractions ranged from 102.29 to 444.87 mg/100 g dm in the stems and leaves, respectively, 8-to-9-fold higher than the values obtained in the present study [26].

The flowers of *S. alpina* were distinguished by their anthocyanin content and significant presence of flavones and flavonols (F&F), whereas the leaves exhibited the highest concentrations of polyphenolic compounds, including phenolic acids, PhGs, and procyanidin polymers. In contrast, the stems and roots were characterized by lower concentrations of the analyzed compounds. This compound distribution variability highlights each plant part’s distinct metabolic specialization and suggests their differentiated potential applications. The obtained results, supported by data from the literature, indicate substantial interspecies variation and differences in the quantity and quality of polyphenolic groups and individual compounds. These findings emphasize the necessity for a detailed phytochemical analysis of *S. alpina* components to better understand their pharmacological potential and functional properties.

### 2.3. Pro-Healthy Properties

#### Enzyme Inhibition Potency

The enzymatic inhibitory activity of extracts obtained from different organs of *S. alpina* against key digestive enzymes—α-amylase, α-glucosidase, and pancreatic lipase—revealed significant differences in their inhibitory potential. These enzymes play a crucial role in carbohydrate and lipid metabolism by breaking them down into simpler forms, such as fatty acids, monosaccharides, and carbohydrate oligomers [26,48,49]. The study results suggest the potential application of *S. alpina* extracts in the regulation of metabolic disorders such as type 2 diabetes and obesity, by limiting the activity of enzymes responsible for carbohydrate and lipid digestion. Lower IC_50_ values indicate stronger enzyme inhibition, which is a beneficial effect for dietary therapies aimed at glycemic and lipid control [49,50,51,52].

The highest inhibitory activity against α-amylase and α-glucosidase was observed in extracts derived from stems, suggesting their potential role in controlling postprandial hyperglycemia, a critical factor in the prevention and management of type 2 diabetes [53]. In *S. palustris*, the highest inhibitory activity was found in the leaves and flowers, with IC_50_ values of 6.85 and 8.14 mg/mL for α-amylase and 12.71 and 11.20 mg/mL for α-glucosidase, respectively [26]. Likewise, methanolic extracts of *S. byzantina* K.Koch and *S. iberica* subsp. *iberica* var. *densipilosa* exhibited IC_50_ values of 0.31 and 0.34 mg/mL for α-amylase and 1.95 and 6.14 mg acarbose/g extract for α-glucosidase [54]. Additionally, the ability to inhibit α-amylase in extracts from *S. cretica* subsp. *mersinaea, S. cretica* subsp. *smyrnaea,* and *S. cretica* subsp. *kutahyensis* was 418.6, 61.5, and 315.5 mg acarbose/g extract, respectively, whereas the α-glucosidase inhibitory activity for *S. cretica* subsp. *mersinaea* and *S. cretica* subsp. *smyrnaea* was 734.5 and 47.84 mg acarbose/g extract, respectively [42,43,44]. Moreover, extracts from *S. iberica* subsp. *iberica var. densipilosa* and *S. byzantina* exhibited α-amylase inhibitory activity of 219.5 and 200.1 mg acarbose/g extract, respectively [55]. In aqueous extracts of *S. cretica* subsp. *anatolica*, the α-amylase inhibitory activity was 5.12 mg acarbose/g, whereas in methanolic extracts, it reached 471.19 mg acarbose/g [56]. These findings indicate that *S. alpina* extracts, particularly those obtained from stems, exhibit strong inhibitory effects on α-amylase and α-glucosidase, making them potential candidates for managing metabolic disorders such as diabetes and obesity. The variation in enzyme inhibition among different plant organs highlights their diverse phytochemical composition and bioactive potential, suggesting that further research should focus on identifying specific compounds responsible for these inhibitory effects.

The analysis of pancreatic lipase activity revealed that flower extracts exhibit moderate inhibitory effects, suggesting their potential role in limiting fat digestion and supporting weight control. This mechanism may contribute to reducing obesity risk by decreasing lipid absorption, a phenomenon previously confirmed in studies on plant extracts with similar properties [26,56]. When comparing the obtained results with available data for *S. palustris*, leaf extracts of this species exhibited 1.7-fold stronger inhibitory effects against pancreatic lipase than the inhibition observed in *S. alpina* stem extracts [26]. Until now, no reports have addressed the ability of extracts from different organs of *S. alpina* to inhibit digestive enzyme activity. The current findings suggest that extracts of this plant could be significant in the prevention and supportive treatment of metabolic diseases, particularly through the limitation of enzymatic digestion of carbohydrates and lipids.

Furthermore, a comparative analysis with other *Stachys* species revealed considerable variation in inhibitory activity, which may result from differences in phytochemical composition, extraction methods, and the polarity of solvents used. Future research should focus on identifying the specific compounds responsible for inhibitory activity and assessing their potential bioavailability under physiological conditions [26].

The assessment of antioxidant activity in different parts of *S. alpina* using the ABTS and FRAP assays revealed statistically significant differences (*p* > 0.05) in their capacity to neutralize free radicals and their reducing potential [Table 3]. The results clearly indicate that leaves and flowers exhibit the highest antioxidant activity, expressed in Trolox equivalents per gram of dry mass, which correlates strongly with their elevated polyphenolic content. This phenomenon underscores their essential role in protection against oxidative stress, a finding consistent with previous studies on other medicinal plant species [26,41,50,52].

Compared to leaves and flowers, roots and stems demonstrated 2- and 5-fold lower antioxidant activity in the ABTS assay, confirming their limited ability to neutralize reactive oxygen species (ROS). A similar pattern was observed in the FRAP assay, where the reducing activity (Fe^3+^ to Fe^2+^ reduction) in roots and stems was 5- and 14-fold lower, respectively, compared to the values recorded for leaves and flowers. These findings align with previous studies on *S. palustris*, where the highest antioxidant activity was also observed in leaves and flowers, whereas stems and roots displayed significantly lower ROS-neutralizing capacity [26]. Moreover, a comparative analysis of the antioxidant activity of different components of *S. palustris* and *S. aplina* demonstrated that the ABTS- and FRAP-measured antioxidant capacities in leaves, flowers, stems, and roots of *S. palustris* were on average 1.3-fold (ABTS) and 1.4-fold (FRAP) lower than in *S. aplina* [26].

The observed differences in antioxidant activity among the various organs of *S. alpina* are strongly correlated with their polyphenolic content, which aligns with previous reports in the literature. Leaves and flowers, as essential generative and photosynthetic structures, accumulate higher amounts of flavonoids and phenolic acids, explaining their superior ability to neutralize free radicals and reduce transition metal ions [26,57,58,59,60].

The Fe^3+^ → Fe^2+^ reducing power varies significantly across different *Stachys* species. For *S. anisochila* Vis. & Pancic, *S. backeana*, *S. plumosa* Griseb., and *S. alpina* ssp. *dinarica*, the recorded values were 1.87, 1.83, 0.46, and 1.36 μmol Fe^2+^/mg dry matter (dm), respectively, significantly lower than the reducing power of L-ascorbic acid (7.41 μmol Fe^2+^/mg dm) [61]. For *S. byzantina* and *Stachys iberica* subsp. *iberica* var. *densipilosa*, these values were reported as 48.12 and 85.35 mg Trolox equivalents (TE)/g extract [54]. In comparison, the antioxidant activity determined by the ABTS method for *S. tmolea* was 32.3 mg TE/g dm, while the FRAP assay yielded 41.9 mg TE/g dm [47]. Similarly, for *S. byzantina* and *Stachys iberica* subsp. *iberica* var. *densipilosa*, the ABTS and FRAP values were reported as 111.02 and 138.16 mg TE/g extract, respectively [54]. Extracts from the whole plants of *S. cretica* subsp*. kutahyensis* Akçiçek, *S. cretica* subsp. *mersinaea,* and *S. cretica* ssp*. anatolica* exhibited ABTS radical scavenging activities of 175.8, 292.7, and 112.2 mg TE/g dm extract, respectively, while their iron-reducing capacities were measured at 239.1, 236.4, and 127.2 mg TE/g dm extract [42,43,44,56].

Despite the lack of direct comparability with the results obtained for *S. alpina*, these data highlight that antioxidant activity is highly dependent on solvent type, solvent purity, polarity, extraction procedures, fractionation methods, and the bioactivity of the compounds present in specific plant organs [26].

### 2.4. Multivariate Analysis

**Figure 3:**
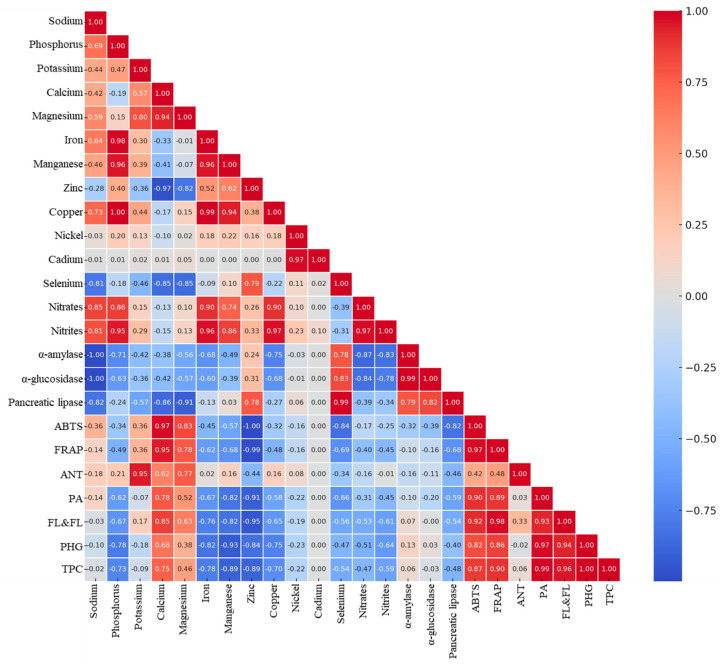
Pearson correlation. Legend: PA, phenolic acid; ANTs, anthocyanins; TPCs, total phenolic compounds; F&Fs, flavonols and flavones; PHGs, phenylethanoid glycosides.

The principal components from the PCA (82.6%) explain a significant portion of the variance in the dataset, indicating clear differences in the chemical composition of individual organs (Figure 3). PC1 (46.2%) reflects major differences in the content of macro- and microelements, as well as in enzymatic activity. PC2 (38.4%) is primarily associated with antioxidant levels and digestive enzyme activity. The sample distribution in the PCA plot shows a distinct grouping of leaves and flowers, suggesting a similar chemical profile, with a markedly higher content of polyphenols and antioxidant compounds (FRAP, ABTS). In contrast, the roots and stems cluster separately, which suggests a dominance of metals (Fe, Mn, Zn, Cu) and stronger inhibition of pancreatic lipase activity. Similar observations between the fractions of *S. palustris* and the analyzed components were reported by Lachowicz-Wiśniewska et al. [26] while examining the principal components of PCA. The study highlighted that hydrolyzed tannins and polymeric procyanidins were the predominant metabolites in leaves and flowers (PC1), whereas phenylethanoid glycosides (PhGs) dominated in roots (PC2) [26]. Additionally, Bouasla et al. [18] observed similar relationships while analyzing the leaves of *S. marrubiifolia*.

**Figure 4:**
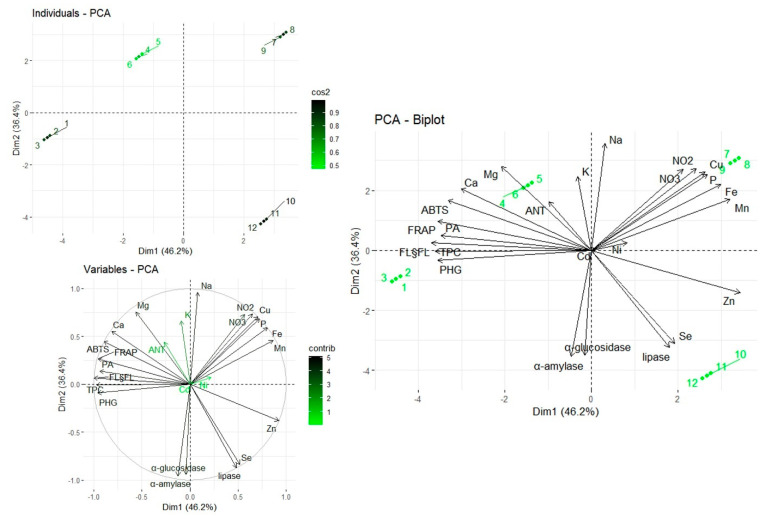
Principal component analysis (PCA) of all components of leaves, flowers, stalks, and roots of *S. alpina L.* Legend: 1–3, duplicate results for leaves; 4–6, duplicate results for flowers; 7–9, duplicate results for stems; 10–12, duplicate results for roots; Na, sodium; Mg, magnesium; K, potassium; P, phosphorus; Ca, calcium; Fe, iron; Zn, zinc; Cu, copper; Mn, manganese; Se, selenium; Ni, nickel; Cd, cadmium; PA, phenolic acid; ANTs, anthocyanins; TPCs, total phenolic compounds; F&Fs, flavonols and flavones; PHGs, phenylethanoid glycosides; lipase, pancreatic lipase.

The correlation relationships between chemical components provide additional insights into the role of individual elements and metabolites in plant function (Figure 4). It was found that high concentrations of nitrogen, phosphorus, and potassium in the leaves promote the synthesis of secondary metabolites, such as flavonoids and polyphenols, which exhibit antioxidant and anti-inflammatory properties. Calcium and magnesium, which dominate in the stems and roots, may support the nervous and muscular systems, while also stabilizing enzymes.

Microelements, including iron, manganese, and zinc, show a strong correlation with enzymatic activity, suggesting their role in plant metabolism and potential biological properties. α-Amylase and α-glucosidase exhibit a negative correlation with metal content, indicating that their higher concentrations may inhibit the activity of these enzymes. The opposite relationship is observed for lipase, whose activity increases with rising concentrations of iron, manganese, and zinc. A positive correlation between nitrate content and digestive enzyme activity suggests that these compounds may influence carbohydrate metabolism. The analysis of relationships between antioxidant activity and polyphenol content confirms that the ABTS and FRAP assays exhibit a strong correlation with total polyphenol content (R^2^ = 0.87 and 0.90, respectively), indicating the high antioxidant potential of this plant. It is well known that the synergistic action of bioactive compounds contributes to antioxidant activity, which primarily depends on their concentration [26,47]. Additionally, anthocyanins exhibit a moderate correlation with antioxidant activity (R^2^ABTS = 0.42 and 0.48), while the correlation for other polyphenol groups ranges from R^2^ = 0.82 (ABTS: PhGs) to 0.98 (FRAP: F&Fs). A strong correlation between polyphenols and ABTS and FRAP tests has also been reported by Lachowicz-Wiśniewska et al. [26] and Bahadori et al. [42,43] in studies analyzing the correlation of bioactive compounds in *S. palustris* and *S. cretica* subsp. *mersinaea*.

The ability to inhibit digestive enzyme activity is strongly correlated with selenium content, while pancreatic lipase inhibition is correlated with zinc (R^2^ = 0.78). Moreover, both the total polyphenol content and specific polyphenol classes are strongly positively correlated with calcium and magnesium, while anthocyanins additionally correlate with potassium. A strong correlation is also observed between mineral components, such as magnesium with calcium and potassium and iron/manganese with phosphorus (R^2^ = 0.98/0.96). Furthermore, a strong relationship between certain elements and polyphenols was reported by Bouasla et al. [18] in their study on leaves of *S. marrubiifolia*. The study indicated that copper, an element involved in enzyme biosynthesis and activation, was correlated with phenols, particularly biphenols, orthoquinones, or benzoxepins [18]. Additionally, manganese may promote chromium degradation [62].

## 3. Materials and Methods

### 3.1. Plant Materials

*S. alpina* was collected in Poland and, more precisely, in the area of Szczytna (53°33′46″ N 20°59′07″ E), Lower Silesia, Poland. The plant was collected randomly in August from different parts of a field (the total cultivation area was 1 ha). Samples were collected from plants aged 3 years; each organ (flowers, leaves, stems, roots) was sampled in triplicate (*n* = 3; each 1 kg). The root, leaves, stems, and flowers of the *S. alpina* plant were identified by Professor Jan Oszmiański. The fresh flowers, leaves, stems, and roots were directly frozen at −25 °C, and then freeze-dried and ground. The powders were kept frozen (−25 °C) until planned analysis at around 2 weeks. A sample of each of these collections is stored in the laboratory (J.O.) for further reference.

### 3.2. Determination of Micro- and Macroelements and Heavy Metals

The contents of elements in the roots, leaves, stems, and flowers of *S. alpina* L. were assessed after mineralization: P, K, Ca, Mg were tested after wet mineralization in H_2_SO_4_ (96%) and HClO_4_ (70%); Fe, Mn, Zn, Cu, Se, Ni, Cd were measured after mineralization in HNO_3_ (65%) and HClO_4_ (70%) in a ratio of 3:1.

The concentration of K was tested by atomic emission spectrometry; the concentrations of K, Ca, Mg, Fe, Mn, Zn, Cu, Se, Ni, and Cd were tested by flame atomic absorption spectroscopy using iCE 3000 Series (Thermo Fisher Scientific, Bishop Meadow Road, Loughborough, UK); and the content of P was measured by the colorimetric method on a Specol 221 apparatus (Carl Zeiss, Germany) [63,64].

### 3.3. Determination of Polyphenolic Compounds

Extraction was performed as described by Lachowicz-Wiśniewska et al. [65]. Briefly, plant material was mixed with 50% ethanol (*v*/*v*) in a plant material-to-solvent ratio of 1:10 (w/v), at room temperature. The extraction was performed twice by incubation for 15 min under sonication (Sonic 6D, Polsonic, Warsaw, Poland) and with occasional shaking. Next, the slurry was centrifuged at 19,000× *g* for 10 min, and the supernatant was filtered through a hydrophilic PTFE 0.20 μm membrane (Millex Samplicity Filter, Merck) and used for analysis. The determination of phenolic compounds was performed by UPLC-PDA-ESI-MS/MS (Waters Corporation, Milford, MA, USA), in accordance with the protocol described by Lachowicz-Wiśniewska et al. [65]. The identification was performed at the following wavelengths: phenolic acids at 320 nm, flavonols at 360 nm, anthocyanins at 520 nm, and flavan-3-ols at 280 nm. Separation of individual polyphenols was conducted using a UPLC BEH C18 column (Waters Corporation, Milford, MA, USA) at 30 °C. Individual polyphenolic standards were acquired from Merck (Darmstadt, Germany). The detection of polyphenolic compounds was exercised under positive ionization. The calibration curves were run at 360 nm for the standard of apigenin 7-glucoside and chrysoeriol; at 320 nm for the standard of chlorogenic acid, caffeic acid, *p*-coumaric acid, sinapic acid, caffeoylquinic acid, verbascoside, actenoside, stachysoside B, and allsonoside; at 520 nm for the standard of cyanidin 3*-O-*glucoside and peonidin-3*-O-*glucoside; and at 280 nm for the standard of (−) epicatechin and (+)-catechin, at concentrations ranging from 0.05 to 5 mg/mL (*R*^2^ = 0.9998), purchased from Sigma-Aldrich (Poznań, Poland). The results are given as mg/100 g dm.

### 3.4. Determination of Procyanidins

The determination of phloroglucinolysis was performed in accordance with the protocol described by Lachowicz et al. [66]. Briefly, the material was extracted with 0.8 mL of methanolic phloroglucinol-ascorbic acid solution and 0.4 mL of methanolic HCl (0.3 M), incubated at 50 °C for 30 min with vortexing. The reaction was stopped on ice, diluted with sodium acetate buffer, centrifuged (20,000× *g*, 10 min, 4 °C), and stored at 4 °C prior to analysis. Separation of phloroglucinolysis products was carried out using the Cadenza CD C18 column (Imtakt, Japan). Calibration curves were established using (+)-catechin and (−)-epicatechin-phloroglucinol adduct standards. The results are given as mg/100 g dm.

### 3.5. Determination of Enzyme Inhibition Potency

α-amylase, α-glucosidase, and pancreatic lipase were selected due to their critical roles in carbohydrate and lipid digestion, making them relevant for metabolic syndrome and obesity management. Protocols for the determination of anti-diabetic activity, such as α-amylase and α-glucosidase inhibitory activity, and anti-obesity activity, such as the lipase inhibitory effect of samples, have already been described by Ochmian et al. [64], Nakai et al. [67], Podsędek et al. [68], and Nickavar et al. [69]. The salivary α-amylase activity in the samples was detected qualitatively and quantitatively, using the Phadebas^®^ amylase assay (Magle Life Science, Lund, Sweden), by gel diffusion evaluation and absorbance measurement at 620 nm (*n* = 4), respectively. Using a fixed assay time, the absorbance reading gives the amylase activity of the solution via a standard curve provided with each kit. The α-glucosidase activity was monitored using an enzyme kit (KT, Sigma-Aldrich, Inc., St. Louis, MO, USA): the Lipase Activity Assay Kit—Sigma-Aldrich. The results of antioxidant activity are given as IC_50_ values (mg of powder material per 1 mL of reaction mixture under assay conditions).

### 3.6. Determination of Antioxidant Activity

Methods for determining anti-radical cation activity (ABTS) and ferric-reducing power (FRAP) were applied in our study as reported by Re et al. [70] and Benzie and Strain [71]. The extraction was prepared as described by Lachowicz et al. [72]. The results of antioxidant activity are given as mmol Trolox/g d.w.

### 3.7. Statistical Analysis

Statistica 12.5 (StatSoft, Kraków, Poland) was used for statistical analyses, including one-way ANOVA and Tukey’s HSD test to assess for significant differences (*p* ≤ 0.05) between organs of *S. alpina*. The material was collected in 3 replicates, and each replicate was made in 3 batches, with a total *n* = 9. Multivariate analysis was performed by applying principal component analysis (PCA) and Pearson’s correlations.

## 4. Limitations and Practical Implications

Despite the promising findings regarding the polyphenolic profile, mineral composition, and biological activities of *S. alpina*, several limitations must be acknowledged. First, the biological activity of plant extracts was assessed in vitro only, which does not fully account for the complexity of metabolic processes occurring in living organisms. The lack of in vivo studies limits the ability to extrapolate the results to physiological conditions, including the absorption, distribution, metabolism, and excretion of the bioactive compounds. Additionally, no toxicity evaluation was conducted for the plant extracts, which is a critical factor when considering future applications in nutraceuticals or pharmaceuticals.

Future studies should include animal model experiments and long-term toxicity assessments to validate the safety and efficacy of *S. alpina* extracts in the context of chronic disease prevention. Moreover, clinical studies would be required to confirm their therapeutic potential in humans.

Nonetheless, the study provides a strong foundation for the use of *S. alpina* as a natural source of enzymatic inhibitors and antioxidants, potentially applicable in the development of functional foods, dietary supplements (e.g., capsules or powders), or even phytopharmaceuticals aimed at managing metabolic disorders such as obesity and type 2 diabetes.

## 5. Conclusions

This study demonstrated that *S. alpina* is a valuable source of polyphenolic compounds and essential minerals, particularly its leaves and flowers, which exhibited the highest antioxidant capacity and enzyme inhibitory potential. The identified bioactive compounds, including verbascoside and chlorogenic acid, as well as high levels of calcium, magnesium, zinc, and manganese, contribute to the plant’s ability to modulate oxidative stress and inhibit α-amylase, α-glucosidase, and pancreatic lipase activities. These findings suggest that *S. alpina* holds promising potential for use in the development of natural therapeutics aimed at preventing or managing metabolic disorders. However, further research involving in vivo studies and toxicity assessments is necessary to confirm the safety, bioavailability, and therapeutic efficacy of these plant-derived extracts.

## Figures and Tables

**Table 1:** The content of macro- and microelements in different parts of *S. alpina*.

Group	Element	Leaves	Flowers	Stalk	Root
Macroelements [mg/kg]	N	25,300.00 ± 50.60 d **	7400.00 ± 14.80 a	18,700.00 ± 37.40 d	15,400.00 ± 30.80 b
	P	21,000.00 ± 42.00 d	12,200.00 ± 24.40 b	15,900.00 ± 31.80 d	7200.00 ± 14.40 a
	K	10,100.00 ± 20.20 b	8600.00 ± 17.20 a	13,600.00 ± 27.20 b	8600.00 ± 17.20 a
	Ca	5400.00 ± 10.80 b	2200.00 ± 4.40 a	10,100.00 ± 20.20 b	9200.00 ± 18.40 c
	Mg	2900.00 ± 5.80 a	1800.00 ± 3.60 a	3900.00 ± 7.80 c	3100.00 ± 6.20 b
Microelements [mg/kg]	Fe	89.43 ± 0.18 c	56.17 ± 0.11 b	61.10 ± 0.12 b	34.67 ± 0.07 b
	Mn	155.30 ± 0.31 c	107.67 ± 0.22 b	114.33 ± 0.23 b	32.30 ± 0.06 a
	Zn	16.37 ± 0.03 c	21.00 ± 0.04 d	7.73 ± 0.02 b	5.37 ± 0.01 a
	Cu	12.60 ± 0.03 d	6.23 ± 0.01 b	8.67 ± 0.02 c	3.37 ± 0.01 a
	Se	<0.01	<0.01	<0.01	<0.01
	Ni	<0.01	<0.01	<0.01	<0.01
	Cd	0.03 ± 0.001 b	0.42 ± 0.001 c	0.02 ± 0.001 ab	0.01 ± 0.001 a
NO_3_⁻ [mg/kg]		109.00 a	187.50 b	552.30 c	102.00 a
NO_2_⁻ [mg/kg]		0.70 a	1.20 b	1.80 c	0.90 a

** Values are means ± standard deviation. *n* = 9; a–d: means ± SD followed by different letters within same line represent significant differences (*p* < 0.05).

**Table 2:** Quality and quantity of polyphenolic compounds in leaves, flowers, stalks, and roots of *S. alpina* [mg/100 g dm].

No	λ [nm]	MS-MS [M-H]−/[M-H]+	Compounds	Rt * [min]	*S. alpina* L.			
					Flowers	Leaves	Stems	Roots
1	320	461/315	Decaffeoyl-acetoside	3.67	53.13 ± 1.06 b **	92.65 ± 1.85 a	28.07 ± 0.56 c	15.94 ± 0.32 d
2	325	353/191/179/135	1-Caffeoylquinic acid	3.73	19.83 ± 0.40 b	29.04 ± 0.58 a	18.63 ± 0.37 c	8.26 ± 0.17 d
3	325	353/179	3-Caffeoylquinic acid	3.87	6.70 ± 0.13 c	13.03 ± 0.26 a	11.17 ± 0.22 b	3.42 ± 0.07 d
4	320	461/315	Decaffeoyl-acetoside	4.01	9.57 ± 0.19 c	13.42 ± 0.27 b	14.41 ± 0.29 a	9.37 ± 0.19 cd
5	329	353/294/274/179	Caffeoylquinic acid	4.19	12.87 ± 0.26 b	54.83 ± 1.10 a	4.58 ± 0.09 c	3.60 ± 0.07 d
6	335	339/179	Methoxycinnamic acid hexoside	4.31	6.34 ± 0.13 b	22.75 ± 0.46 a	3.74 ± 0.07 c	nd
7	325	341/179	Caffeoylglucose	4.43	4.45 ± 0.09 b	24.96 ± 0.50 a	2.11 ± 0.04 c	nd
8	329	339/179	Methoxycinnamic acid hexoside	4.63	8.42 ± 0.17 b	36.77 ± 0.74 a	3.9 ± 0.08 c	nd
9	324	353/191/179	5-Caffeoylquinic acid	4.87	1573.15 ± 31.46 b	3277.83 ± 65.56 a	1221.95 ± 24.44 c	590.35 ± 11.81 d
10	517	449/287	Cyanidin 3-glucoside	5.05	31.33 ± 0.63 a	nd	nd	nd
11	277	459/283	Palustrinoside	5.10	2.59 ± 0.05 d	11.46 ± 0.23 a	9.37 ± 0.19 b	8.92 ± 0.18 c
12	323	397/179	5-Sinapoylquinic acid	5.34	85.19 ± 1.70 a	64.73 ± 1.29 b	4.30 ± 0.09 d	6.50 ± 0.13 c
13	326	353/191/179	4-Caffeoylquinic acid	5.50	52.3 ± 1.05 b	157.20 ± 3.14 a	27.32 ± 0.55 c	nd
14	517	535/463/301	Peonidin-3*-O-*glucoside	5.80	9.59 ± 0.19 a	nd	nd	nd
15	311	337/191	3*-O-p*-Coumaroylquinic acid	5.91	33.71 ± 0.67 b	96.70 ± 1.93 a	5.46 ± 0.11 c	2.00 ± 0.04 d
16	330	623/461/443/315	Verbascoside	6.02	2.74 ± 0.05 d	22.87 ± 0.46 a	11.63 ± 0.23 b	8.53 ± 0.17 c
17	324	785/161	Betonyoside E	6.16	16.29 ± 0.33 b	21.08 ± 0.42 a	11.65 ± 0.23 c	nd
18	517	535/287	Cyanidin 3-malonylglucoside	6.27	163.27 ± 3.27 a	nd	nd	nd
19	331	785/161	Betonyoside E	6.28	11.71 ± 0.23 c	41.60 ± 0.83 a	8.04 ± 0.16 d	15.83 ± 0.32 b
20	330	785/161	Betonyoside E isomer	6.33	23.38 ± 0.47 b	31.09 ± 0.62 a	4.22 ± 0.08 c	4.06 ± 0.08 d
21	329	785/161	Betonyoside E isomer	6.40	22.67 ± 0.45 a	20.17 ± 0.40 b	2.71 ± 0.05 d	4.35 ± 0.09 c
22	307	337/191	3*-O-p*-Coumaroylquinic acid	6.43	21.29 ± 0.43 b	38.60 ± 0.77 a	1.67 ± 0.03 c	nd
23	328	785/161	Betonyoside E isomer	6.75	12.55 ± 0.25 a	6.97 ± 0.14 b	4.61 ± 0.09 c	nd
24	325	623/461/161	Acteoside	6.81	6.94 ± 0.14 a	2.60 ± 0.05 b	2.41 ± 0.05 b	nd
25	516	549/301	Peonidin- 3-malonylglucoside	6.94	10.86 ± 0.22 a	nd	nd	nd
26	334	665/623/503/461/443/299/284	Chryseriol 7*-O-*[6‴*-O-*acetyl]-allosyl(1→2)glucoside	7.02	34.43 ± 0.69 a	29.27 ± 0.59 b	nd	nd
27	334	785/623/161	Echinacoside-glucoronoid	7.14	8.98 ± 0.18 b	21.99 ± 0.44 a	2.97 ± 0.06 c	nd
28	330	785/623/161	Echinacoside isomer	7.21	9.42 ± 0.19 c	42.83 ± 0.86 a	10.09 ± 0.20 b	nd
29	331	785/623/161	B-OH-Forsythoside B methylether	7.29	16.95 ± 0.34 d	58.97 ± 1.18 a	24.62 ± 0.49 c	33.73 ± 0.67 b
30	328	755/623/593/161	Stachysoside A	7.40	886.73 ± 17.73 c	3022.8 ± 60.46 a	892.96 ± 17.86 b	690.72 ± 13.81 d
31	328	623/461/315	Verbascoside	7.59	3072.07 ± 61.44 b	4618.88 ± 92.38 a	681.49 ± 13.63 d	1170.71 ± 23.41 c
32	274/305/326	609/285	Isoscutellarein-7*-O-*allosyl(1→2)]-glucoside	7.70	nd	120.84 ± 2.42 a	nd	nd
33	347	623/447/285	Kaempferol hexose glucuronide	7.72	126.13 ± 2.52 a	98.6 ± 1.97 b	nd	nd
34	329	755/623/461/161	Forsythoside B	7.79	15.11 ± 0.30 c	77.89 ± 1.56 a	11.48 ± 0.23 d	19.84 ± 0.4 b
35	330	623/461/161	Isoverbascoside	7.91	219.21 ± 4.38 d	1025.28 ± 20.51 a	250.56 ± 5.01 c	297.27 ± 5.95 b
36	350	609, 447, 285	Luteolin 7*-O-*dihexoside	8.01	29.62 ± 0.59 b	88.40 ± 1.77 a	13.55 ± 0.27 c	8.87 ± 0.18 d
37	326	623/461/161	Acteoside	8.06	102.33 ± 2.05 b	316.88 ± 6.34 a	39.79 ± 0.8 d	81.61 ± 1.63 c
38	326	693/651/609/489/471/429/285	Luteolin 7*-O-*[6‴*-O-*acetyl]-allosyl-(1→2)-[6′′*-O-*acetyl]-glucoside	8.14	25.86 ± 0.52 b	58.95 ± 1.18 a	13.67 ± 0.27 c	nd
39	329	769/593/161	Allysonoside	8.34	195.22 ± 3.9 b	260.35 ± 5.21 a	122.08 ± 2.44 d	164.81 ± 3.30 c
40	317	769/593/315/161	Allysonoside	8.39	55.06 ± 1.10 c	100.55 ± 2.01 b	121.23 ± 2.42 a	nd
41	329	675/593/447/285	Isoscutellarein-3-glucoside-rhamnoside	8.59	109.33 ± 2.19 a	61.21 ± 1.22 b	33.14 ± 0.66 d	59.44 ± 1.19 c
42	344	755/623/593/461	Samioside	8.78	nd	7.98 ± 0.16 a	1.86 ± 0.04 b	nd
43	329	461/299	Chrysoeriol-glucoside	8.86	51.79 ± 1.04 b	65.23 ± 1.30 a	34.26 ± 0.69 d	37.86 ± 0.76 c
44	324	635/593/461/431/269	apigenin 7*-O-*[6‴*-O-*acetyl]-allosyl(1→2)glucoside	8.91	148.31 ± 2.97 a	93.2 ± 1.86 b	10.20 ± 0.20 d	18.08 ± 0.36 c
45	324	515/353/191	3,4-Dicaffeoylquinic acid	8.92	147.51 ± 2.95 a	89.12 ± 1.78 b	7.92 ± 0.16 d	16.96 ± 0.34 c
46	335	635/593/461/431/269	apigenin 7*-O-*[6‴*-O-*acetyl]-allosyl(1→2)glucoside	9.01	48.21 ± 0.96 b	223.23 ± 4.46 a	7.18 ± 0.14 c	7.07 ± 0.14 c
47	324	515/353/191/179	3,5-Dicaffeoylquinic acid	9.04	99.47 ± 1.99 a	64.21 ± 1.28 b	1.31 ± 0.03 d	2.81 ± 0.06 c
48	347	665/623/299/284	Chryseriol 7*-O-*[6‴*-O-*acetyl]-allosyl(1→2)glucoside	9.06	138.21 ± 2.76 a	71.16 ± 1.42 b	3.96 ± 0.08 d	6.45 ± 0.13 c
49	347	665/299	Chrysoeriol-7*-O-*[6‴-acetylallopyranosyl-(1 → 2)]-glucopyranoside (=stachyspinoside)	9.18	421.86 ± 8.44 b	454.35 ± 9.09 a	106.94 ± 2.14 d	132.26 ± 2.65 c
50	330	665/623/461/299	Chryseriol 7*-O-*[6‴*-O-*acetyl]-allosyl(1→2)glucoside	9.36	10.33 ± 0.21 a	5.84 ± 0.12 b	3.82 ± 0.08 c	1.79 ± 0.04 d
51	329	783/607/475/329	Leonoside B	9.49	30.41 ± 0.61 d	125.83 ± 2.52 a	53.91 ± 1.08 b	36.12 ± 0.72 c
52	329	623/461/299/284	4*-O-*Methylisoscutellarein-7*-O-*[allosyl-(1→2)]-glucopyranoside	9.59	10.72 ± 0.21 b	16.16 ± 0.32 a	nd	9.50 ± 0.19 c
53	349	783/607/475/329	Leonoside B	9.69	38.02 ± 0.76 a	18.16 ± 0.36 b	2.27 ± 0.05 c	2.36 ± 0.05 c
54	329	651/429/285	Isoscutellarein-7*-O-*[6‴-acetyl-allopyranosyl-(1 →2)]-glucopyranoside	9.85	40.87 ± 0.82 b	143.98 ± 2.88 a	15.99 ± 0.32 d	20.84 ± 0.42 c
55	323	665/623/299/284	4′*-O-*methylisoscutellarein 7*-O-*[6‴*-O-*acetyl]-allosyl(1→2)glucoside	10.30	11.3 ± 0.23 b	71.66 ± 1.43 a	5.40 ± 0.11 d	5.92 ± 0.12 c
56	324	515/353/191	4,5-Dicaffeoylquinic acid	11.07	2.24 ± 0.04 b	10.9 ± 0.22 a	1.09 ± 0.02 c	0.21 ± 0.001 d
57	347	707/665/647/503/299	4′*-O-*methylisoscutellarein 7*-O-*[6‴*-O-*acetyl]-allosyl-(1→2)-[6′′*-O-*acetyl]-glucoside	11.26	0.99 ± 0.02 c	52.34 ± 1.05 a	3.33 ± 0.07 b	0.48 ± 0.01 cd
58	347	473/269	Apigenin hexoside acetyl derivative	11.80	0.19 ± 0.001 c	10.43 ± 0.21 a	0.34 ± 0.01 b	0.13 ± 0.001 c
			Degree of polymerization		1.64 ± 0.03 b	1.94 ± 0.04 a	1.16 ± 0.02 c	1.14 ± 0.02 d

* Rt, retention time in minutes; ** Values are means ± standard deviation. *n* = 9; a–d means ± SD followed by different letters within same line represent significant differences (*p* < 0.05).

**Table 3:** The value of pro-healthy parameters.

Parts of *S. aplina*	α-Amylase IC_50_ [mg/mL]	α-Glucosidase IC_50_ [mg/mL]	Pancreatic Lipase IC_50_ [mg/mL]	ABTS mmol TE/g dm	FRAP mmol TE/g dm
Leaves	27.77 ± 0.56 **c	32.73 ± 0.65 c	51.28 ± 1.03 c	19.30 ± 0.39 a	7.62 ± 0.15 a
Flowers	25.31 ± 0.51 b	31.33 ± 0.63 b	45.31 ± 0.91 a	16.85 ± 0.34 b	6.65 ± 0.13 b
Stalks	20.07 ± 0.40 a	24.88 ± 0.50 a	50.78 ± 1.02 b	9.70 ± 0.19 c	1.44 ± 0.03 c
Roots	33.16 ± 0.66 d	40.60 ± 0.81 d	88.75 ± 1.77 d	3.74 ± 0.07 d	0.46 ± 0.01 d

** Values are means ± standard deviation. *n* = 9; a–d means ± SD followed by different letters within same line represent significant differences (*p* < 0.05).

## Data Availability

Data are available from the corresponding author.
